# Supplementary material for: Metagenomic binning with assembly graph embeddings
Source: Bioinformatics. 2022 Aug 16;38(19):4481–7. doi: 10.1093/bioinformatics/btac557 (PMC9525014; doi:10.1093/bioinformatics/btac557)
Supplement: btac557_supplementary_data [file btac557_supplementary_data.zip › amber outputs.zip!/amber outputs/strong100/genome/contamination.pdf]

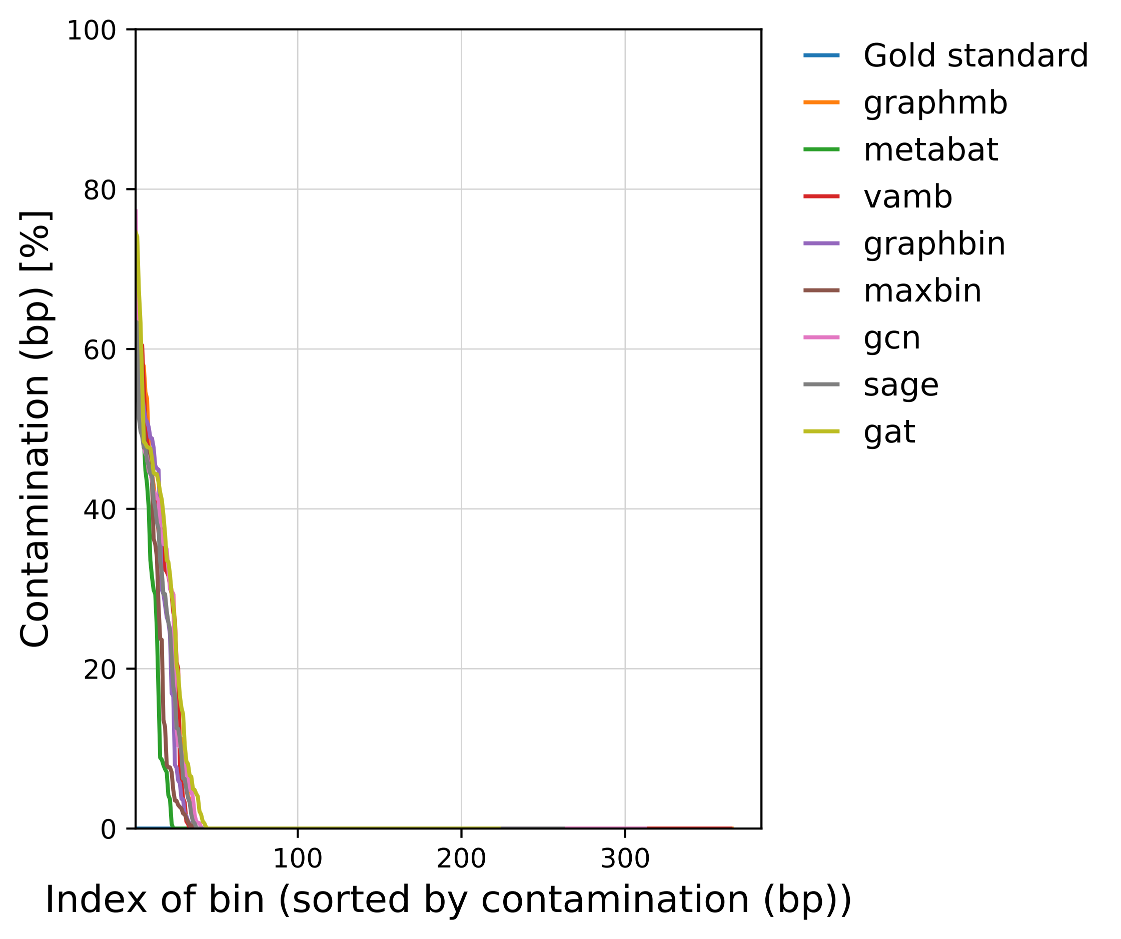
Contamination (bp) [%]
0
20
40
60
80
100
100
200
300
Index of bin (sorted by contamination (bp))
Gold standard
graphmb
metabat
vamb
graphbin
maxbin
gcn
sage
gat
